# Intracellular Localization of the Proteins Encoded by Some Type II Toxin-Antitoxin Systems in Escherichia coli

**DOI:** 10.1128/mBio.01417-21

**Published:** 2021-08-03

**Authors:** Alexander Mager, Tommy Safran, Hanna Engelberg-Kulka

**Affiliations:** a Department of Microbiology and Molecular Genetics, IMRIC, The Hebrew University–Hadassah Medical School, Jerusalem, Israel; University of British Columbia

**Keywords:** *E. coli*, toxin-antitoxin, protein localization

## Abstract

Bacterial toxin-antitoxin (TA) systems encode a toxin and an antitoxin that counteracts the toxin. Such TA systems are found abundantly on bacterial chromosomes and on extrachromosomal genetic elements. The toxin is always a protein. Based on the nature of the antitoxin (protein or RNA) and on their mode of regulation, they are classified into six groups (I to VI). In the group II TA systems, both the toxin and the antitoxin are proteins, and the gene specifying the antitoxin precedes the gene specifying for the toxin. Here, we studied the intracellular localization in Escherichia coli cells of the proteins specified by the following type II TA modules: *mazEF*, *chpBIK*, *mqsRA*, and *rnlAB*. We visualized the localization of these proteins by fusing them with the fluorescent protein mCherry using recombinant DNA technology. We used fluorescence microscopy and image analysis software to obtain and quantify protein distribution data. With the exception of the *chpBIK* TA module, we found that the localization of each toxin-antitoxin complex was different from the localization of the toxin itself. Our results demonstrate clearly that the presence of the antitoxin shifts the localization of its respective toxin toward the middle of the cell, which could contribute to the reduction of cellular toxicity.

## INTRODUCTION

Bacterial toxin-antitoxin (TA) systems are unique genetic elements that consist of pairs of genes, one encoding a toxin and one encoding an antitoxin. These systems are found abundantly, either on extrachromosomal elements, such as plasmids and bacterial phages, or on the bacterial chromosome itself. To date, toxin-antitoxin systems are divided into six types (1). The most studied are the type II TA systems. They share the following characteristics (2): (i) the TA system harbors two adjacent genes; (ii) the product of one is a stable and toxic protein, whereas the product of the other is an unstable protein that antagonizes the toxic effect of the first by physical interaction; (iii) the toxin and antitoxin proteins are coexpressed; (iv) mostly, the antitoxin protein is encoded by the upstream gene in the module; (v) the toxin and antitoxin proteins interact; and (vi) the antitoxin protein is degraded by a specific bacterial protease (2). Here, we focus on only to type II TA systems.

The first chromosomal TA system discovered, and, to date, the most studied, is the Escherichia coli *mazEF* module (3). The *mazEF* module encodes the antitoxin MazE and the toxin MazF (3). The *mazEF* system is triggered by various stressful conditions (4), which include (i) extreme amino acid starvation, leading to the production of the starvation signaling molecule guanosine-3′,5′-bispyrophosphate (ppGpp), which is synthesized by the *relA*-encoded protein (RelA) (3); (ii) inhibition of transcription and/or translation by antibiotics, including rifampin, chloramphenicol, and spectinomycin (5); (iii) DNA damage caused by thymine starvation (6), as well as by mitomycin C, nalidixic acid, and UV irradiation (4); (iv) oxidative stress (H_2_O_2_) (4); and (v) high temperature (50°C) (4). All of these stressful conditions prevent the expression of the labile protein MazE. When *mazEF* is not expressed, the existing labile antitoxin MazE is degraded by the ATP-dependent ClpAP serine protease; therefore, there is nothing to interfere with the activity of the stable toxin MazF (3). It has also been shown that MazE and MazF interact in the form of a linear heterohexamer composed of alternating MazF and MazE homodimers (7). MazF has been described as a sequence-specific endoribonuclease that cleaves single-stranded mRNAs at either the 3′ or the 5′ side of the first A in ACA sequences (8).

Homologous to *mazEF* is the E. coli chromosomal system *chpBIK.* This system consists of a pair of genes, *chpBI* and *chpBIK*, which encode the antitoxin ChpBI and the toxin ChpBK, respectively (9). The toxin ChpBK consists of 116 amino acid residues, and its sequence shows 35% identity and 52% similarity to that of MazF. Like MazF, ChpBK is a sequence-specific endoribonuclease. It has been reported that, both *in vivo* and *in vitro*, ChpBK cleaves mRNAs at either the 5′ or the 3′ side of the A residue in ACY sequences (where Y represents U, A, or G). This cleavage of mRNAs in the E. coli cells leads to a reduction of protein synthesis (10). However, in contrast to the activation of E. coli MazF, activation of the toxin ChpBK does not cause cell death (10).

Another member of the type II TA systems is the *mqsRA* toxin-antitoxin module. MqsR, the toxin partner of the *mqsRA* system, is a sequence-specific endoribonuclease that cleaves GCU sites in mRNA (11). The induction of MqsR causes cell growth arrest; coinduction of the MsqA antitoxin leads to recovered cell growth (11). The observation that the genes specifying biofilm formation lack GCU sequences suggested that the toxin MqsR may have a role in E. coli biofilm formation (11). In fact, such a role has been shown in the soil bacterium Pseudomonas putida (12).

A recently discovered type II TA system is the *rnlAB* toxin-antitoxin module. The toxin RnlA is part of a larger protein, RNase LS, which is responsible for rapid degradation of mRNAs. RnlA is essential for its endonuclease activity (13). LS RnlA is involved in phage T4 development by cleaving late mRNAs of the phage at 3′ to G sites (13). Cells that have a mutation in the RnlA component accumulate high levels of rRNA (14). The RnlA toxin is inhibited by the labile antitoxin RnlB, which is degraded by ClpXP protease and Lon (15).

Here, we studied the intracellular localization of several representatives of the type II TA systems in E. coli cells. In order to visualize the localization of these proteins, we fused them with the fluorescent protein mСherry using recombinant DNA technology. Using fluorescence microscopy techniques and Image-Pro software, we examined the intracellular localization of the protein products of the type II TA systems *mazEF*, *chpBIK*, *mqsRA*, and *rnlAB*.

## RESULTS

### The *mazEF* toxin-antitoxin module.

Initially, we studied the intracellular localization of the protein products of the *mazEF* TA system. In all of the experiments reported here, we exclusively used E. coli MC4100 *relA^+^* cells. Since the concentrations of the toxin-antitoxin proteins are extremely low, we established their localization by the use of the fluorescent protein mCherry located on the low-copy-number plasmid pBAD18. The proteins were induced by 10 mM arabinose. At the first stage, we determined the intracellular localization of mCherry itself (unfused). This was done in E. coli MC4100 *relA^+^* cells with the *mcherry* gene, located on plasmid pBAD18 and induced by arabinose. As can be seen in Fig. S1 in the supplemental material, mCherry itself is dispersed in the cytoplasm, i.e., it is not specifically localized in E. coli MC4100 *relA^+^* cells.

10.1128/mBio.01417-21.2 FIG S1 Intracellular localization of mCherry in the Escherichia coli MC4100 *relA^+^* strain. mCherry is dispersed homogeneously in the cells. Download FIG S1, TIF file, 2.9 MB. Copyright © 2021 Mager et al. 2021 Mager et al. https://creativecommons.org/licenses/by/4.0/ This content is distributed under the terms of the Creative Commons Attribution 4.0 International license.

To determine the intracellular localization of the protein products of the *mazEF* toxin-antitoxin module in E. coli cells, we fused each of these products (the toxin *mazF*, the antitoxin *mazE*, and the combined *mazEF*) to the fluorescent reporter mCherry. For this purpose, primers were generated to produce *mazF*, *mazE*, and *mazEF* genes lacking stop codons and fused to a linker of three alanine residues, as described in Materials and Methods. The fused genes thus generated were first amplified by PCR and then cloned into the pBAD18-*mcherry* plasmid. The primers used for cloning are listed in the supplemental material. The cloning protocols are described in detail in Materials and Methods. The constructed fusions are illustrated schematically in Fig. S2 in the supplemental material.

10.1128/mBio.01417-21.3 FIG S2 A schematic representation of the fusions constructed in this work. In the cases of *mazE-mcherry*, *mazF-mcherry*, *chpB-mcherry*, *chpBK-mcherry*, *mqsR-mcherry*, *mqsA-mcherry*, *rnlA-mcherry*, and *rnlB-mcherry* fusions, the gene of interest lacking a stop codon is fused to *mcherry* through a 3-alanine linker. In the cases of *mazEF-mcherry* and *chpBIK-mcherry* fusions, the gene sequences of *mazF* and *chpBK*, lacking their respective stop codons, are fused with *mcherry* through a linker of 3 alanines. Download FIG S2, TIF file, 1.2 MB. Copyright © 2021 Mager et al. 2021 Mager et al. https://creativecommons.org/licenses/by/4.0/ This content is distributed under the terms of the Creative Commons Attribution 4.0 International license.

**FIG 1:**
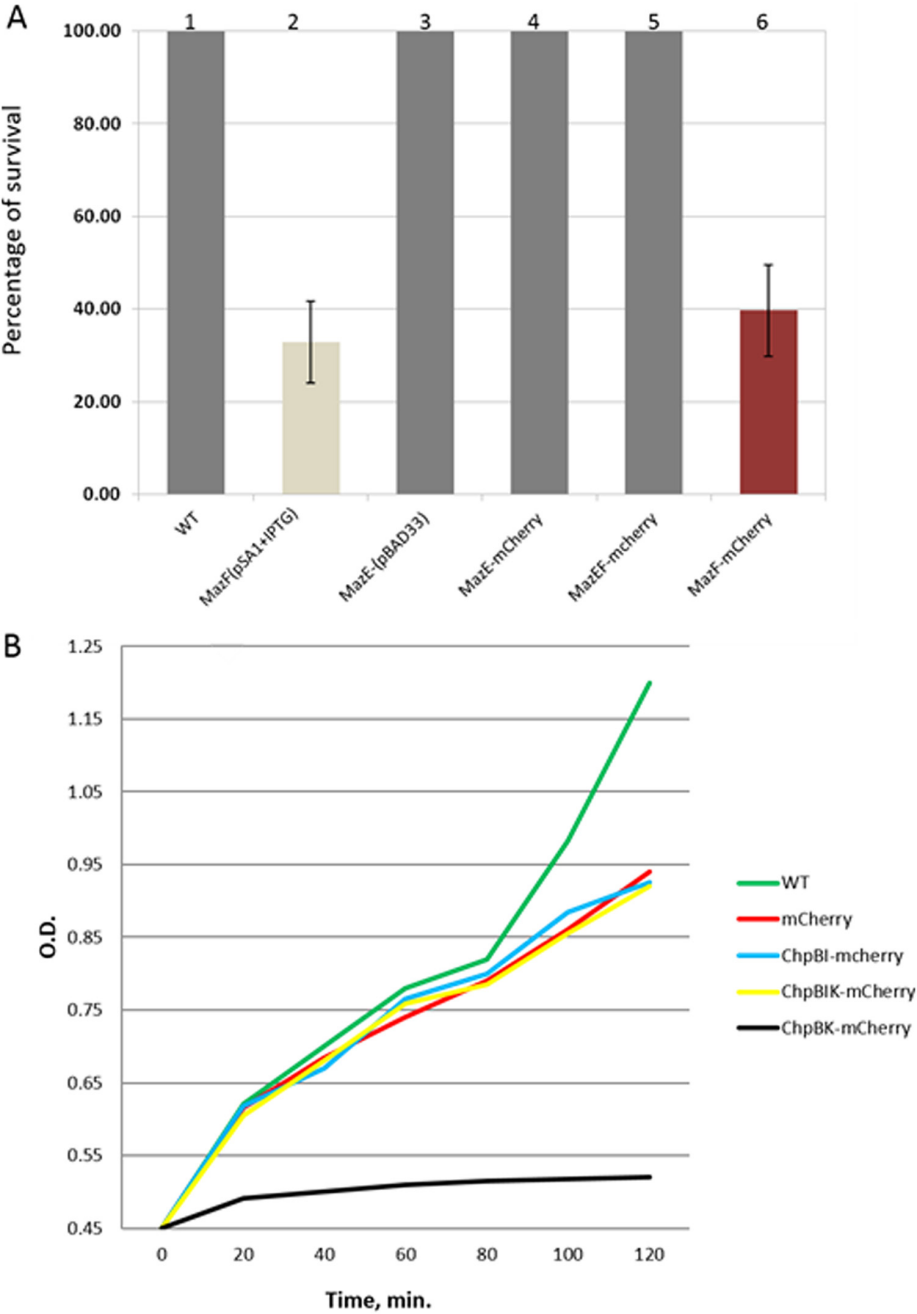
Functionality of the protein products of the *mazEF* and *chpBIK* TA modules fused with mCherry. (A) Effect on bacterial viability of the overproduction of the proteins of the *mazEF* toxin-antitoxin module. Overproduction of MazF-mCherry caused a loss in cell viability (lane 6). This result for MazF-mCherry is equivalent to the result for overproduced unfused MazF (lane 2). No viability loss was observed in the cases of the overproduced antitoxin MazE-mCherry (lane 4) or the combined antitoxin and toxin MazEF-mCherry (lane 5). (B) The effects on the cell growth of the overproduced *mcherry* fusions of the proteins of the *chpBIK* TA module. Induction of ChpBK-mCherry by 10 mM arabinose inhibited cell growth. There was no effect on cell growth as a result of induction of the antitoxin ChpBI or the combined toxin-antitoxin ChpBK by 10 mM arabinose.

A series of assays were performed to determine the effect of the overproduced protein products of the fusions on cell viability. E. coli MC4100 *relA^+^* cells carrying the pBAD18 plasmids with the constructed fusions were grown as described in Materials and Methods. After induction with 10 mM arabinose, cell viability was determined. Figure 1A shows the effect of the overproduced mCherry fusions of the protein products of the *mazEF* TA module on bacterial viability. Overproduction of the MazF-mCherry fusion caused loss of cell viability (Fig. 1A, lane 6), corresponding to the effect of the overproduced MazF that was not fused to mCherry (Fig. 1A, lane 2). On the other hand, no loss of viability was observed in the case of overproduced antitoxin MazE-mCherry (Fig. 1A, lane 4) or the combined antitoxin-toxin MazEF-mCherry (Fig. 1A, lane 5). Thus, fusing mCherry to the protein products of the *mazEF* module did not impair their functionality; even after fusion with mCherry, MazF remains toxic and MazE prevents its toxicity.

**FIG 2:**
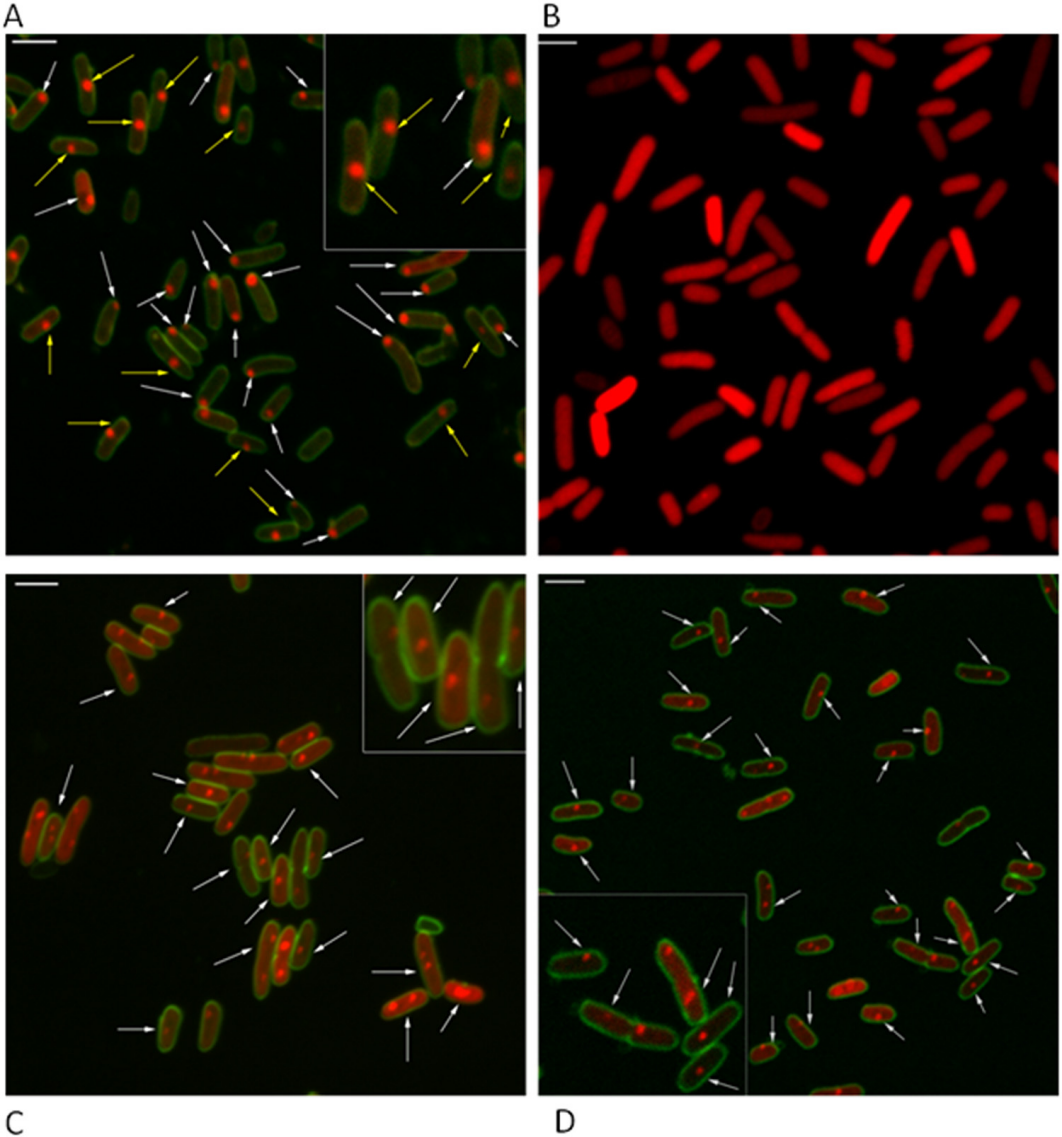
The intracellular localization of the protein products of the *mazEF* TA module in Escherichia coli. (A) Two localizations of the toxin MazF were observed, polar (indicated by white arrows) and nonpolar (indicated by yellow arrows). (B) The antitoxin MazE is dispersed in the cells. (C) MazEF (in which antitoxin MazE and toxin MazF are combined) is mostly localized in the middle of the cells (indicated by white arrows). (D) In the presence of the overexpressed antitoxin MazE, the toxin MazF is mostly localized in the middle of the cells (indicated by white arrows). Cell membranes were labeled with FM 1-43FX (green). Bar, 2 μm. Magnified (2×) sections of the images are shown at the corners.

Figure 2A shows the intracellular localization of the toxin MazF fused with mCherry in the E. coli MC4100 *relA^+^* strain. As can be clearly seen, MazF-mCherry was observed in two distinctly different intracellular localization patterns. There are cells in which MazF-mCherry is located at the poles of the cells (indicated by white arrows). In fact, in 20 of 37 cells of this representative image (which shows one out of 15 similar experiments), MazF-mCherry was localized at the pole of the E. coli cells (Fig. 2A). On the other hand, in the same representative image, in 17 cells MazF-mCherry is not localized at the pole of the cell (indicated by yellow arrows). In contrast, unlike MazF-mCherry, MazE-mCherry, the antitoxin MazE fused with mCherry, did not form foci but was dispersed homogenously in the cell (Fig. 2B).

In the MazEF-mCherry fusion, the *mazF* stop codon is not present. Instead, MazF is fused to a linker of 3 alanine residues followed by mCherry. Thus, the toxin MazF is the mCherry-fused protein, and the overproduced antitoxin MazE is combined with MazF. Unexpectedly, we found MazEF-mCherry to be localized completely differently from either MazF-mCherry or MazE-mCherry. While MazF-mCherry was localized mainly at the cell poles (Fig. 2A) and MazE-mCherry was dispersed throughout the cell (Fig. 2B), MazEF-mCherry was localized mostly in the center of the cell (indicated by the white arrows in Fig. 2C, which represents one of 15 similar experiments).

**FIG 3:**
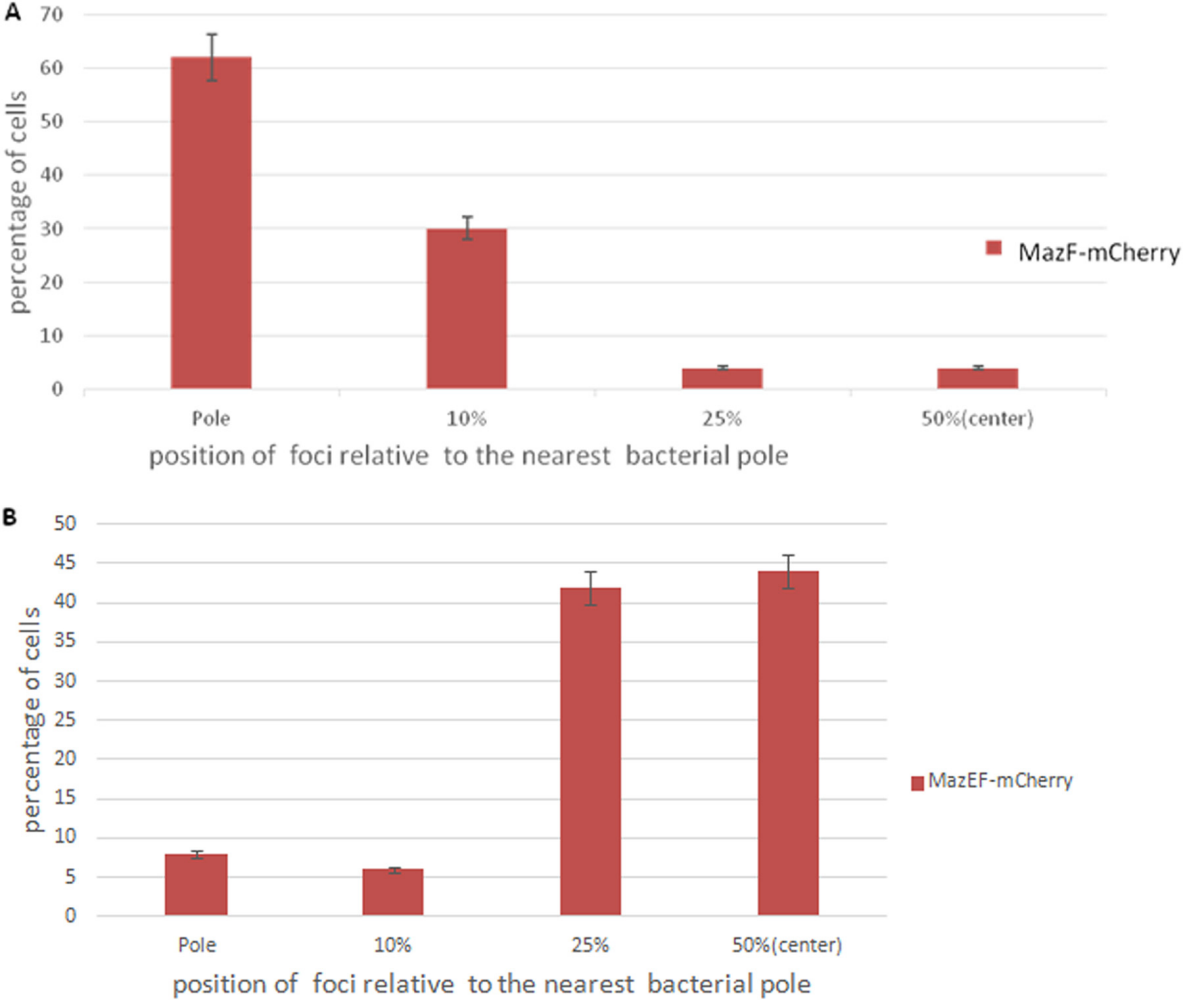
Calculated distribution of the protein products of the *maZEF* TA module in E. coli. The position of each MazF-mCherry focus was calculated as a ratio of the distance of the focus to the nearest pole of the cell. (A) The toxin MazF is mostly localized at the poles of the cells. (B) The combined toxin-antitoxin MazEF is mostly localized at the midcell.

In order to establish the preferred localization pattern of the toxin MazF by itself, and of the combined toxin MazF and antitoxin MazE (MazEF) in E. coli MC4100 *relA*^+^ cells, we determined the positions of the foci relative to the nearest pole of the cell. This was done in 250 cells for each of the cases using Image-Pro software. We measured the positions of the foci relative to the nearest pole of the cell and converted these measurements to a percentage of cell length. We divided the cells into groups according to the relative measured positions of the foci. In 62% of the cells, MazF-mCherry foci were localized at the pole (Fig. 3A, lane 1). In the remaining 38% of the cells (Fig. 3A, lanes 2, 3, 4, and 5), MazF-mCherry foci were mostly localized between the pole and the center of the cell.

In contrast to the calculated position of the toxin MazF by itself (Fig. 3A), a completely different pattern of localization was observed when the toxin MazF was combined with the antitoxin MazE. In 86% of the cells, the MazEF-mCherry foci were localized either in the center of the cells or in the area close to the cell centers (Fig. 3B, lanes 4 and 5). On the other hand, only 14% of the foci were localized at the poles or in the area close to the pole (Fig. 3B, lanes 1, 2, and 3).

These experiments were carried out by the induction of *mazEF* proteins with 10 mM arabinose. Similar results were obtained by the use of 5 mM arabinose (see Fig. S3 and S4 in the supplemental material), which was the lowest concentration that permitted their visualization.

10.1128/mBio.01417-21.4 FIG S3 The intracellular localization of MazF-mcherry in the E. coli MC4100 *relA*^+^ strain. The E. coli cells harboring pBAD-MazF-*mcherry* plasmids were grown as described in Materials and Methods, but 5 mM arabinose was used for induction and the incubation time was reduced to 2 h. Download FIG S3, TIF file, 0.9 MB. Copyright © 2021 Mager et al. 2021 Mager et al. https://creativecommons.org/licenses/by/4.0/ This content is distributed under the terms of the Creative Commons Attribution 4.0 International license.

10.1128/mBio.01417-21.5 FIG S4 The intracellular localization of MazEF-*mcherry* in the E. coli MC4100 *relA*^+^ strain. The E. coli cells harboring pBAD-MazEF-*mcherry* plasmids were grown as described in Materials and Methods, but 5 mM arabinose was used for induction and the incubation time was reduced to 2 h. FIG S4, TIF file, 2.9 MB Copyright © 2021 Mager et al. 2021 Mager et al. https://creativecommons.org/licenses/by/4.0/ This content is distributed under the terms of the Creative Commons Attribution 4.0 International license.

To support our discovery that the interaction of MazE with MazF causes a shift in the localization of MazF from the poles toward the center of the E. coli cells, we repeated this experiment with MazF-mCherry and MazE generated from different plasmids. We wished to recreate the situation in which the antitoxin is overexpressed. Here, only the toxin is fused with mCherry, and the antitoxin is overexpressed. For this purpose, we cotransformed the pBAD18-*mazF*-*mcherry* plasmid with a compatible pBAD33*-mazE* plasmid into E. coli MC4100 *relA^+^* cells and examined the localization of MazF-mCherry. Figure 2D demonstrates the intracellular localization in E. coli MC4100 *relA^+^* of MazF-mCherry in the presence of MazE, when the latter is derived from another plasmid. As shown once again, here MazF-mCherry is also not localized at the poles. Rather, it is localized in the center of the cells (indicated by white arrows).

### The *chpBIK* toxin-antitoxin module.

We asked if the localization patterns of the *mazEF* TA system protein products are unique, or whether they are similar to those of its homologue, the *chpBIK* TA system. As we had done for the *mazEF* system, to examine the intracellular localization of the protein products of the *chpBIK* TA system, we fused the fluorescent reporter mCherry to the toxin ChpBK, to the antitoxin ChpBI, and to the combined ChpBIK. Constructed fusions are illustrated schematically in Fig. S2 in the supplemental material.

Unlike the TA module *mazEF*, the TA module *chpBIK* is not involved in cell death, but rather inhibits E. coli cell growth (10). As shown in Fig. 1B, ChpBK-mCherry induction by 10 mM arabinose inhibits cell growth. On the other hand, there is no effect on cell growth when the antitoxin ChpBI is combined with ChpBK. Thus, the fusion with mCherry did not interfere with the functionality of the protein products of the c*hpBIK* system.

**FIG 4:**
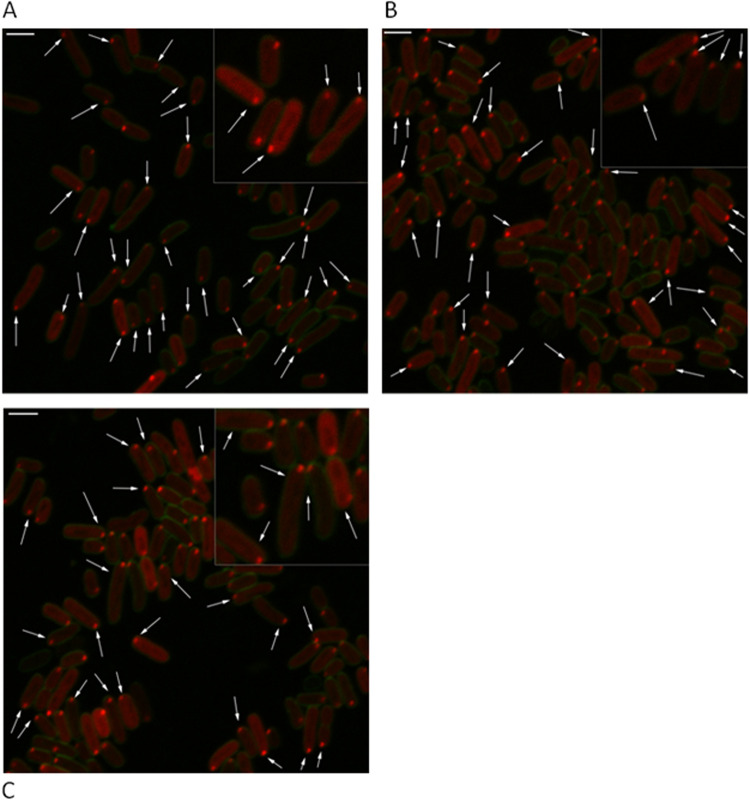
Intracellular localization of the protein products of the *chpBIK* TA module in E. coli. All of the protein products of the *chpBIK* TA module were found localized mostly at the poles of the cells. (A) The toxin ChpBK, (B) the antitoxin ChpB, and (C) the combined toxin-antitoxin ChpBIK. Cell membrane was labeled with FM 1-43FX (green). Bar, 2 μm. Magnified (2×) sections of the images are shown at the corners.

We observed a clear pattern of intracellular localization for the toxin ChpBK-mCherry. ChpBK-mCherry formed foci only at the poles of the cells (Fig. 4A, indicated by white arrows, representing one out of 15 experiments). Similarly, the antitoxin ChpBI-mCherry fusion also formed foci at the poles of the cells (Fig. 4B, indicated by white arrows).

In the toxin-antitoxin ChpBIK-mCherry fusion, the stop codon of *chpBK* was not present; in its place, ChpBK was fused to a linker of three alanine residues followed by mCherry. Thus, the mCherry-fused protein is the toxin ChpBK. In addition to the overproduction of ChpBK-mCherry, ChpBI was also overproduced, generating a combined ChpBIK product in which ChpBK is fused with mCherry. When the toxin ChpBK was combined with the antitoxin ChpBI, the foci were localized at the cell poles (Fig. 4C, indicated by white arrows; one out of 15 typical experiments).

**FIG 5:**
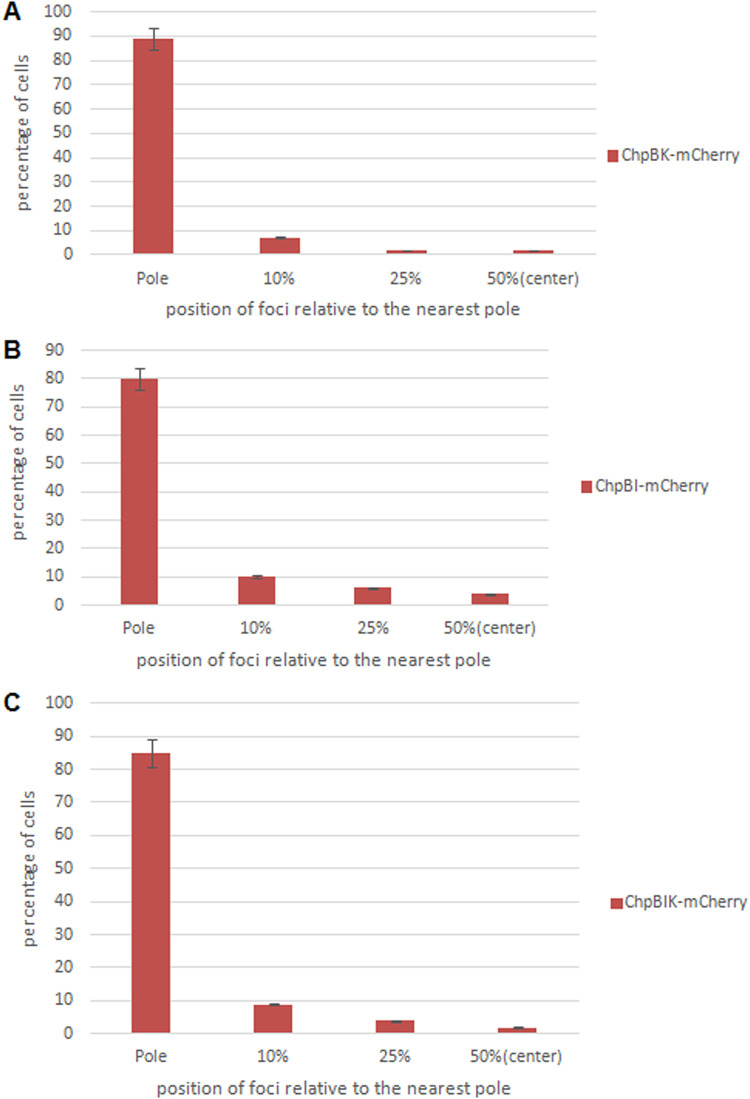
Calculated distribution of the protein products of the *chpBIK* TA module in E. coli. The position of each focus was calculated as a ratio of the distance of the focus to the nearest pole of the cell. The toxin ChpBK (A), the antitoxin ChpB (B), and the combined toxin-antitoxin ChpBIK (C) are mostly localized at the poles of the cells.

As described above for the *mazEF* TA module, we established the cellular localizations of the antitoxin ChpBI, the toxin ChpBK, and the combined toxin-antitoxin ChpBIK by determining the positions of each one relative to the nearest cellular pole. Figure 5 shows the calculated positions of the foci of ChpBK-mCherry, ChpBI-mCherry, and ChpBIK-mCherry in E. coli MC4100 *relA^+^* cells. As can be seen, nearly all of the foci of the protein products of the *chpBIK* toxin-antitoxin module have a clear pattern of localization in E. coli cells. The foci are localized at the pole of the cells. This was true in 86% of the cells in the case of the toxin ChpBK-mCherry (Fig. 5A, lane 1), in 88% of the cells in the case of antitoxin ChpBI-mCherry (Fig. 5B, lane 1), and in 86% of the cells in the case of toxin-antitoxin ChpBIK-mCherry (Fig. 5C, lane 1). Thus, in stark contrast to our results in the case of the interaction of the antitoxin MazE with the toxin MazF, the interaction of the antitoxin ChpBI with the toxin ChpBK did not cause a shift in the localization of the toxin ChpBK from the poles toward the centers of the cells.

### The *mqsRA* toxin-antitoxin module.

Having found that the protein products of the *mazEF* and *chpBIK* TA modules had different intracellular localization patterns, we asked if other type II TA modules might each have unique intracellular localization patterns. First, we considered the *mqsRA* toxin-antitoxin module, generating mCherry fusions of the toxin MqsR and the antitoxin MqsA as we did for the *mazEF* and *chpBIK* modules. The primers used for cloning are listed in the supplemental material, and the constructed fusions are illustrated schematically in Fig. S2 in the supplemental material.

**FIG 6:**
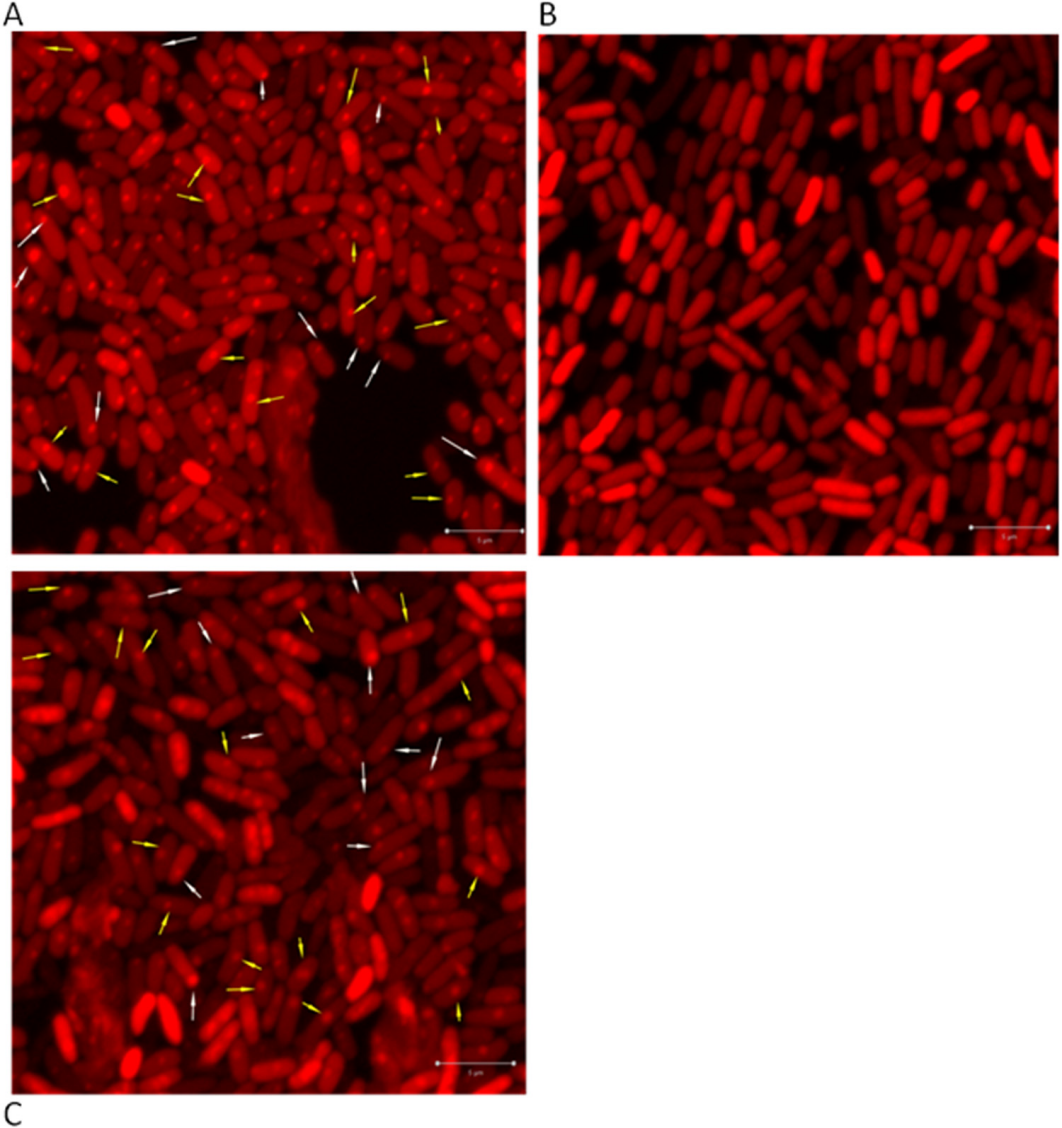
Intracellular localization of the protein products of the *mqsRA* TA module in E. coli MC4100 *relA^+^.* (A) The toxin MqsR is localized between the pole and the middle (indicated by white arrows) and in the middle of the cell (indicated by yellow arrows). (B) The antitoxin MqSA is dispersed in the cell. (C) The toxin-antitoxin MqsRA is localized between the pole and the middle (indicated by white arrows) and in the middle of the cell (indicated by yellow arrows). Bar, 5 μm.

Figure 6A demonstrates the intracellular localization of the toxin MqsR fused with mCherry in the E. coli MC4100 *relA^+^* strain. As shown, two main intracellular localization patterns of MqsR were observed. First, in one subpopulation of the cells, MqsR-mCherry was located at the middle of the cell (indicated by yellow arrows). In another subpopulation of the cells, the toxin MqsR was localized between the pole and the middle of the cell (indicated by white arrows).

Figure 6B represents the intracellular localization of the antitoxin MqsA fused with mCherry. In contrast to the toxin MqsR, MqsA-mCherry was dispersed homogenously in the cells.

**FIG 7:**
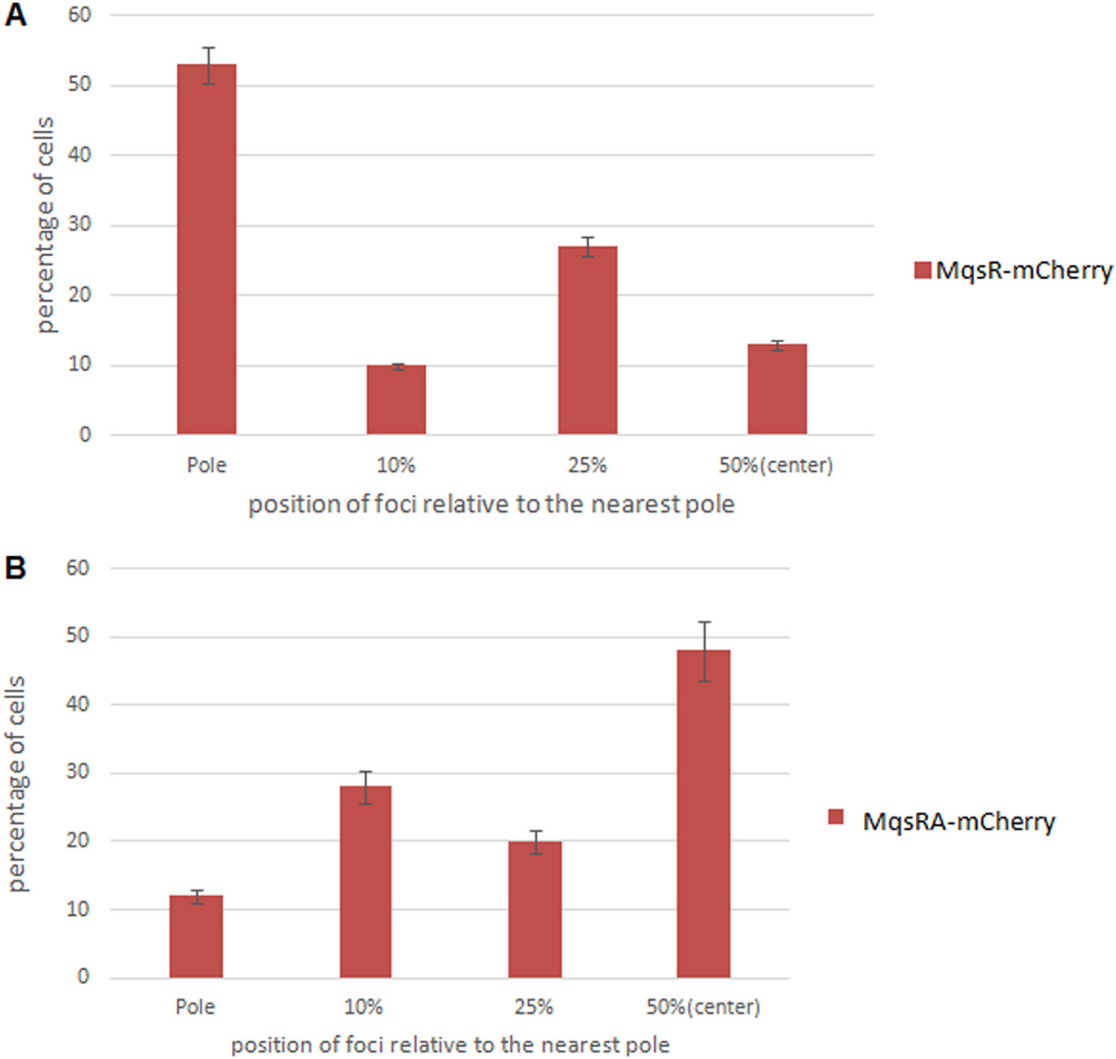
Calculated distribution of the protein products of the *mqsRA* TA module in the E. coli MC4100 *relA^+^* strain. The position of each focus was calculated as a ratio of the distance of the focus to the nearest pole of the cell. (A) The toxin MqsRA is mostly localized between the pole and the midcell. (B) The combined toxin-antitoxin MqsRA is mostly localized at the midcell.

Unlike the *mazEF* and *chpBIK* TA modules, in which the gene for the antitoxin precedes the gene for the toxin, in the *mqsRA* TA module, the *mqsR* toxin gene precedes the *mqsA* antitoxin gene. To recreate the conditions under which the toxin is fused with mCherry and is expressed in the presence of the overproduced antitoxin, we needed to use a different approach than the one described above for the *mazEF* and *chpBIK* modules. We cloned the *mqsA* antitoxin gene to pBAD33 and transformed this newly constructed plasmid into E. coli MC4100 *relA^+^* cells harboring the pBAD-18-*mqsR-mcherry* plasmids. The cloning protocol is described in details in Materials and Methods. Figure 6C shows the intracellular localization of the toxin MqsR-mCherry in the presence of the overproduced antitoxin MqsA in E. coli MC4100 *relA^+^* cells. As shown, there are still two main subpopulations of the cells. In the first, the toxin MqsR is located at the middle of the cell (indicated by yellow arrows). In the second, the toxin MqsR forms foci between the pole and the middle of the cell (indicated by white arrows). Determining the relative distribution of the foci allowed us to clarify the difference between the localization of the toxin MqsR by itself or in the presence of the overproduced antitoxin MqsA (Fig. 7). As shown, the majority (54%) of the MqsR foci were localized between the pole and the middle of the cells (Fig. 7A, lane 2), 34% of the foci were localized in the middle of the cells (Fig. 7A, lane 3), and only 12% were localized at the poles of the cells (Fig. 7A, lane 1). In contrast, when the toxin MqsR was expressed in the presence of the overproduced antitoxin MqsA, most (57%) of the foci were localized the middle of the cells (Fig. 7B, lane 3), 38% between the pole and the middle of the cells (Fig. 7B, lane 2), and only 5% of the foci were localized at the poles of the cells (Fig. 7B, lane 1). Thus, in the presence of the overexpressed antitoxin MqsA, in part of the cells, the toxin MqsR is moved toward the middle of the cell.

### The *rnlAB* toxin-antitoxin system.

Another type II TA system that we investigated was the *rnlAB* toxin-antitoxin module. As described above, we fused mCherry to the toxin RnlA and to the antitoxin RnlB. The primers used for cloning are listed in the supplemental material. The cloning protocols are described in detail in Materials and Methods. The constructed fusions are illustrated schematically in Fig. S2 in the supplemental material.

**FIG 8:**
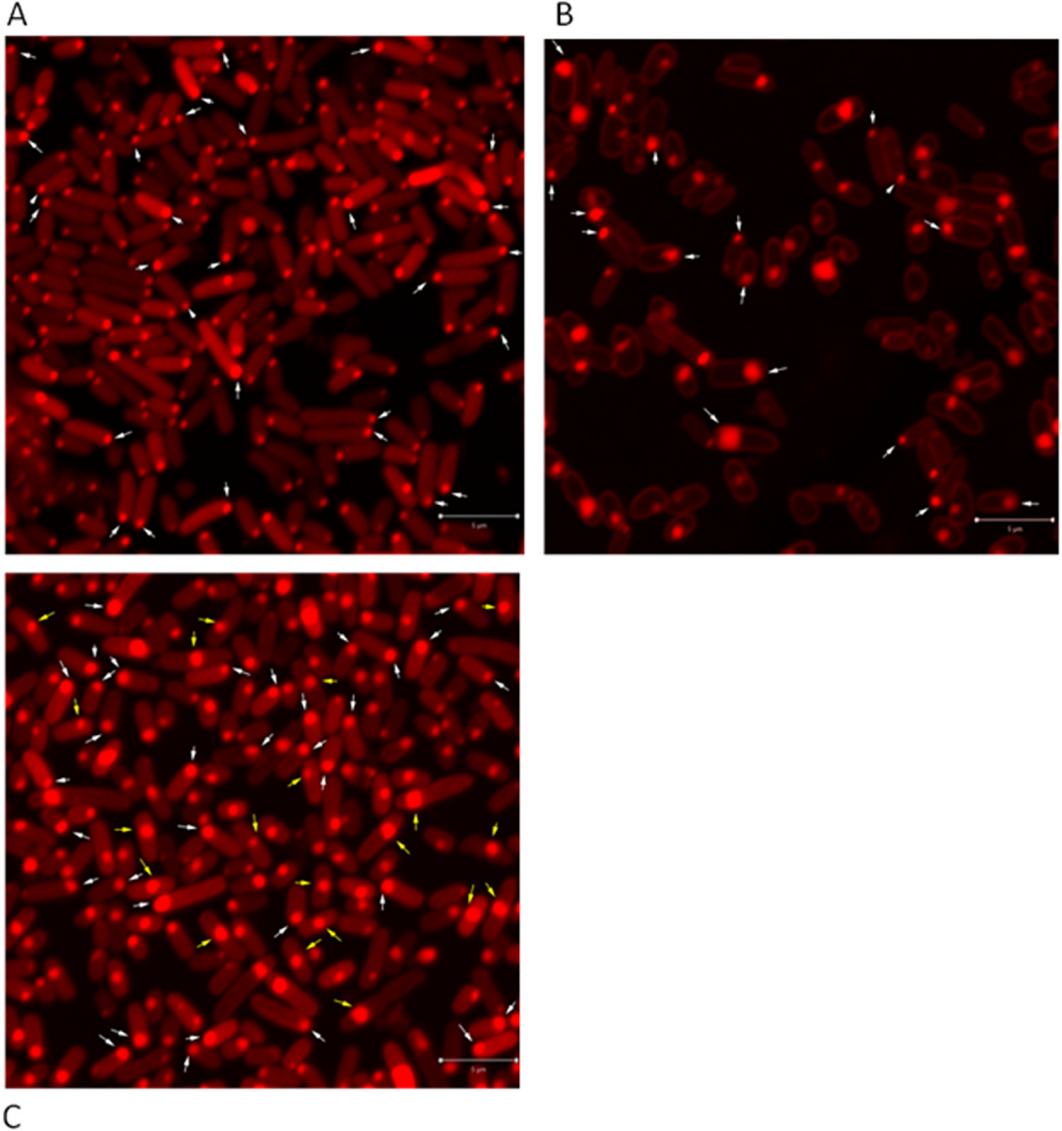
Intracellular localization of the protein products of the *rnlAB* TA module in the E. coli MC4100 *relA^+^* strain. (A) The toxin RnlA is mostly localized at the poles of the cells (indicated by white arrows). (B) The antitoxin RnlB is mostly localized at the poles of the cells (indicated by white arrows). (C) The toxin-antitoxin RnlAB is mostly localized at the poles of the cells; however, a significant portion of the foci are moved toward the middle of the cell (indicated by white arrows).

Figure 8A demonstrates the intracellular localization of the toxin RnlA in E. coli MC4100 *relA*^+^ cells. As can be seen, the toxin RnlA has a clear pattern of cellular localization. RnlA forms foci at the poles of most of the cells (indicated by white arrows). Similarly, the antitoxin RnlB fused with mCherry also formed foci at the poles in most of the cells (Fig. 8B, indicated by white arrows).

**FIG 9:**
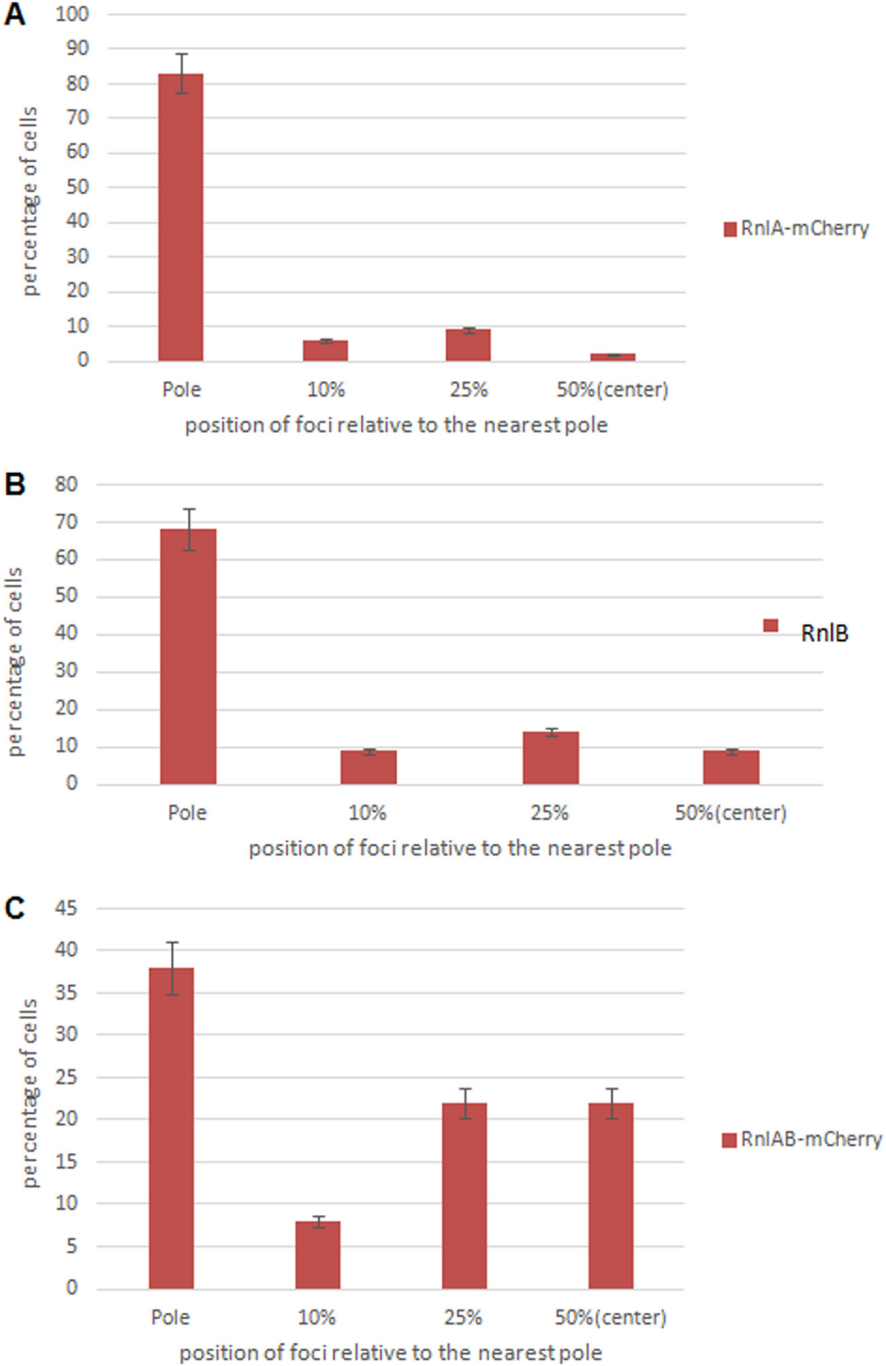
Calculated distribution of the protein products of the *rnlAB* TA module in the E. coli MC4100 *relA^+^* strain. The position of each focus was calculated as a ratio of the distance of the focus to the nearest pole of the cell. (A) The toxin RnlA is localized mostly at the poles of the cells. (B) The antitoxin RnlB is localized at the poles of the cells. (C) The combined toxin-antitoxin RnlAB is localized mostly at the poles of the cells. However, a significant portion of the foci are moved toward the midcell (indicated by yellow arrows).

As in the *mqsRA* TA module, the *rnlA* toxin gene precedes the *rnlB* antitoxin gene, so we repeated the protocol used for the *mqsRA* module for the *rnlAB* system. We cloned the *rnlB* antitoxin gene into pBAD33 and transformed this newly constructed plasmid into cells harboring pBAD-18-*rnlA-mcherry* plasmids. The localization of the toxin RnlA expressed in the presence of the overproduced antitoxin RnlB reveals two major subpopulations of the cells. In one, the toxin RnlA is localized at the poles of the cells (Fig. 8C, indicated by white arrows), and in the second, the toxin RnlA is localized either in the middle of the cell or in the areas close to the middle of the cell (Fig. 8C indicated by yellow arrows). This shift was even more noticeable after we quantified the relative distributions of the RnlA-mCherry foci. The toxin RnlA (Fig. 9A, lane 1) and the antitoxin RnlB (Fig. 9B, lane 1) were mostly localized at the poles of the cells. In contrast, when the toxin RnlA was expressed in the presence of the overproduced antitoxin RnlB, a significant part (40%) of the foci were found not at the poles but rather toward the middle of the cells (Fig. 9C, lanes 2 and 3). Only 60% of the foci are localized at the poles of cells (Fig. 9C, lane 1). Thus, in the case of the *rnlAB* module, as in the cases of the *mazEF* and *mqsRA* modules, the presence of the antitoxin causes the movement of the toxin from the pole toward the center of the cells.

## DISCUSSION

Although bacterial TA systems have been studied extensively (1, 2, 16–18), until now, the localization of their protein products has not yet been investigated at all. Here, we examined the intracellular localization in E. coli of the protein products of four type II TA systems: *mazEF*, *chpBIK*, *mqsRA*, and *rnlAB*. Our observed data from fluorescence microscopy images and our calculated data are summarized in Table 1.

**TABLE 1:** Localization patterns of the protein products of the type II TA systems studied in this work

Protein product	Toxin	Antitoxin	Toxin-antitoxin complex
MazEF	Mostly at the poles	Dispersed	Mostly at the middle of the cell
ChpBIK	Mostly at the poles	Mostly at the poles	Mostly at the poles
MqsRA	Between the pole and the middle of the cell	Dispersed	In some of the cells, the foci are moved towards the middle of the cell
RnlAB	Mostly at the poles	Mostly at the poles	Mostly at the poles; however, a significant part of the focus is moved towards the middle of the cell

### Intracellular localization of the protein products of the *mazEF* toxin-antitoxin module in E. coli cells.

We observed that antitoxin MazE causes a shift in the localization of the toxin MazF from the pole to the middle of the cell.

### Intracellular localization of the protein products of the *chpBIK* toxin-antitoxin module in E. coli cells.

In contrast to the results for the *mazEF* module, each of the protein products of the *chpBIK* TA module, namely the toxin ChpBK, the antitoxin ChpBI, and the combined toxin-antitoxin ChpBIK, is localized at the cell poles.

### Intracellular localization of the protein products of the *mqsRA* toxin-antitoxin module in E. coli cells.

The toxin MqsR is localized either in the region between the pole and the middle of the cell or in the middle of the cells. The antitoxin MqSA is dispersed homogenously in the cells. When the toxin MqsR was expressed in the presence of the antitoxin MqsA, MqsR moved toward the middle of the cells.

### Intracellular localization of the protein products of the *rnlAB* toxin-antitoxin module in E. coli cells.

Both the toxin RnlA and the antitoxin RnlB are localized mostly at the poles of the cells. When the toxin RnlA was expressed in the presence of the antitoxin RnlB, although most of the RnlA molecules remained at the pole of the cells, a significant part of the foci had been moved from the poles toward the middle of the cells.

One could expect that the proteins which belong to the same group of the TA systems (type II) would have the same pattern of the intracellular localization. We were surprised to discover that each of the TA modules studied here had its own pattern of localization for the toxin, the antitoxin, and the toxin-antitoxin complex. However, with the exception of the *chpBIK* TA module, for each TA module, the localization of the toxin-antitoxin complex was different from the localization of the toxin itself. The presence of the antitoxin causes a shift in the localization of the toxin from the pole toward the center of the cells. Thus, we suggest that our results reveal that the antagonistic effect of antitoxins on their cognate toxins might not be based only on their direct structural interactions but may also be caused by changing the intracellular localization of the toxin.

Future experiments should provide explanation of why the toxins tend to migrate toward the poles and the reason for the shift toward the center of the cell by the antitoxin. Does this have anything with the endoribonuclease activity? Interaction with actin-like cytoskeleton proteins? Interaction with ClpXP? In the case of the *mazEF* proteins, they may interact with *mazEF*-specific components, such as the peptide EDF(NNWNN) (19) and components of the stress-induced translation machinery (STM) (20) (see Fig. S5 in the supplemental material).

10.1128/mBio.01417-21.6 FIG S5 MazF is an endoribonuclease cleaving RNA at ACA sites and generates a stress-induced translation machinery (STM). (A) MazF (in red) generates leaderless mRNAs (in green) by cleaving at ACA sites at or closely upstream pf the AUG start codons of some specific mRNAs. (B) MazF also removes the 3′-terminal 43 nucleotides of the 16S rRNA cleaving at an ACA site, thereby removing the anti-Shine-Dalgarno sequence. (C) As a result, an alternative translation machinery is generated, which is responsible for the selective synthesis of specific proteins. Download FIG S5, TIF file, 0.5 MB. Copyright © 2021 Mager et al. 2021 Mager et al. https://creativecommons.org/licenses/by/4.0/ This content is distributed under the terms of the Creative Commons Attribution 4.0 International license.

## MATERIALS AND METHODS

Primers for cloning were obtained from Integrated DNA Technologies (IDT, Hudson, NH). Other materials and suppliers were as follows: PrimeSTAR HS DNA polymerase from TaKaRa, Japan; T4 ligase, Arctic phosphatase, and restriction enzymes XbaI, SacI, EcorI, and HindIII from Thermo Scientific (Epson, Surrey, UK); plasmid miniprep kit from Qiagen (Hilden, Germany); Wizard SV gel and PCR clean-up system from Promega; and FM 1-43FX membrane stain from Thermo Scientific (Epson, Surrey, UK).

### Bacterial strains and plasmids.

We exclusively used the E. coli MC4100 *relA^+^* strain (21) and either pBAD18-*mcherry* (22, 23) or PBAD33 (22) plasmids in which we constructed the protein-mCherry fusions or cloned the genes of the antitoxins (see below).

### Construction of the plasmids.

**(i) pBAD18-mCherry cloning.** The genes encoding the proteins MazE, MazF ChpBI, ChpBK, MqsR, MqsA, RnlA, and RnlB (lacking stop codons) were amplified by PCR from E. coli MC4100 *relA*^+^ genomic DNA. For this purpose, we used the primers that introduced EcoRI (forward primer) and SacI (reverse primer) restriction sites and a gene sequence encoding a three-alanine linker. We used primers that introduced EcoRI (forward primer) and SacI (reverse primer) restriction sites and a gene sequence encoding a linker of three alanine residues. The resulting amplicons were digested with EcoRI and SacI and ligated into pBAD18-*mcherry* plasmids. The following DNA fusions were constructed: *mazE-mcherry*, *mazF-mcherry chpBI-mcherry*, *chpBK-mcherry*, *mqsR-mcherry*, *mqSA-mcherry*, *rnlA-mcherry*, and *rnlB-mcherry.* In the protein products of these DNA fusions, mCherry was fused to the C terminus of the protein of interest through a three-alanine linker. In the case of the combined antitoxin and toxin MazEF-mCherry fusion, the gene sequence of the *mazEF* operon (lacking the stop codon of *mazF*) was amplified by PCR and cloned into pBAD18*-mcherry* plasmid as described above. The following protein products were generated: MazE (by itself without mCherry) and MazF-mCherry, where mCherry was fused to the C terminus of the toxin MazF through a three-alanine linker. In the case of the combined antitoxin and toxin ChpBIK-mCherry fusion, the gene sequence of the *chpBIK* operon (lacking the stop codon of *chpBK*) was amplified by PCR and cloned into the *pBAB18-mcherry* plasmid as described above. The following protein products were generated: ChpBI and ChpBK-mCherry. mCherry was fused to the C terminus of the toxin ChpBK through a three-alanine linker. The primers for cloning and constructed plasmids are listed in the supplemental material.

**(ii) pBAD33 cloning.** In addition to the constructions using pBAD18, gene sequences of *mazE*, *mqsA*, and *rnlB* (lacking stop codons) were amplified by PCR from E. coli MC4100 genomic DNA using the primers that introduced SacI (forward) and XbaI (reverse) sites and a three-alanine linker. The resulting amplicon was cloned into the pBAD33 plasmid. The primers for cloning and constructed plasmids are listed in the supplemental material.

### Growth conditions.

Cloned plasmids were transformed into the E. coli MC4100 *relA*^+^ strain using electroporation. Single colonies were grown overnight in 10 ml of LB medium with 100 μg/ml ampicillin at 37°C and 220 rpm. A 200-μl aliquot of overnight culture was subcultured (1:20 dilution) in 10 ml of LB in the presence of 100 μg/ml ampicillin. The cells were grown at 37°C and 220 rpm to an optical density at 600 nm (OD_600_) of 0.4. To express protein-mCherry fusions, 10 mM arabinose was added to the cell culture, and the cells were incubated at 37°C for 4 h.

### Slide preparation.

Cells were harvested by centrifugation at 14,000 rpm for 2 min and washed twice with phosphate-buffered saline (PBS). For labeling, cell membrane cells were resuspended with 20 μl of FM-143FX dye. Then, for fixation, 100 μl of 4% formalin was applied for 15 min, and the cells were washed twice with PBS (pH 7.2) and resuspended in 10 μl of PBS and 10 μl of polyvinyl alcohol (PVA). Aliquots (10 μl) of resuspended cells were transferred to microscope slides, covered with coverslips, and sealed with nail polish. Slides were dried and stored at −20°C.

### Fluorescent microscopy.

Protein-mCherry fusions were examined using a Zeiss LSM 710 laser scanning microscope with a Plan-Apochromat 63×/1.40 oil lens. mCherry fluorescence was measured as excitation/emission at 561/578 to 696 nm. Images were recorded and analyzed using ZEN 2012 (Zeiss) and Image-Pro Plus 6.0 software (Media Cybernetics, Inc.).

### Viability assays.

Single colonies of the E. coli MC4100 *relA*^+^ strain were grown overnight in 10 ml of M9 medium with 100 μg/ml ampicillin at 37°C, 220 rpm. A 200-μl aliquot of overnight culture was subcultured (1:20 dilution) in 10 ml of LB in the presence of 100 μg/ml ampicillin. The cells were grown at 37°C and 220 rpm to an OD_600_ of 0.4. Protein-mCherry fusions were expressed from pBAD18-*mcherry*-derived plasmids induced by 10 mM arabinose. MazF was expressed from PSA1 plasmid induced by 1 mM isopropyl-β-d-thiogalactopyranoside (IPTG). After 4 h of incubation at 37°C the samples were centrifuged at 14,000 rpm for 2 min. The supernatants were removed, and the pellets were resuspended in 0.5 ml of prewarmed M9 medium. The samples were serially diluted in prewarmed M9, plated on prewarmed LB plates, and incubated at 37°C. The percent survival was determined by dividing the number of colonies obtained from the “experiment” sample by the number of colonies obtained from the MC4100 *relA*^+^ sample used as the control.

10.1128/mBio.01417-21.1 Text S1 Primers used in this work. Download Text S1, DOCX file, 0.01 MB. Copyright © 2021 Mager et al. 2021 Mager et al. https://creativecommons.org/licenses/by/4.0/ This content is distributed under the terms of the Creative Commons Attribution 4.0 International license.

10.1128/mBio.01417-21.7 TABLE S1 Plasmids used in this work. Download Table S1, DOCX file, 0.01 MB. Copyright © 2021 Mager et al. 2021 Mager et al. https://creativecommons.org/licenses/by/4.0/ This content is distributed under the terms of the Creative Commons Attribution 4.0 International license.
