# Supplementary figures and images for: Intracellular Localization of the Proteins Encoded by Some Type II Toxin-Antitoxin Systems in Escherichia coli
Source: mBio. 2021 Aug 3;12(4):e01417-21. doi: 10.1128/mBio.01417-21 (PMC8406201; doi:10.1128/mBio.01417-21)

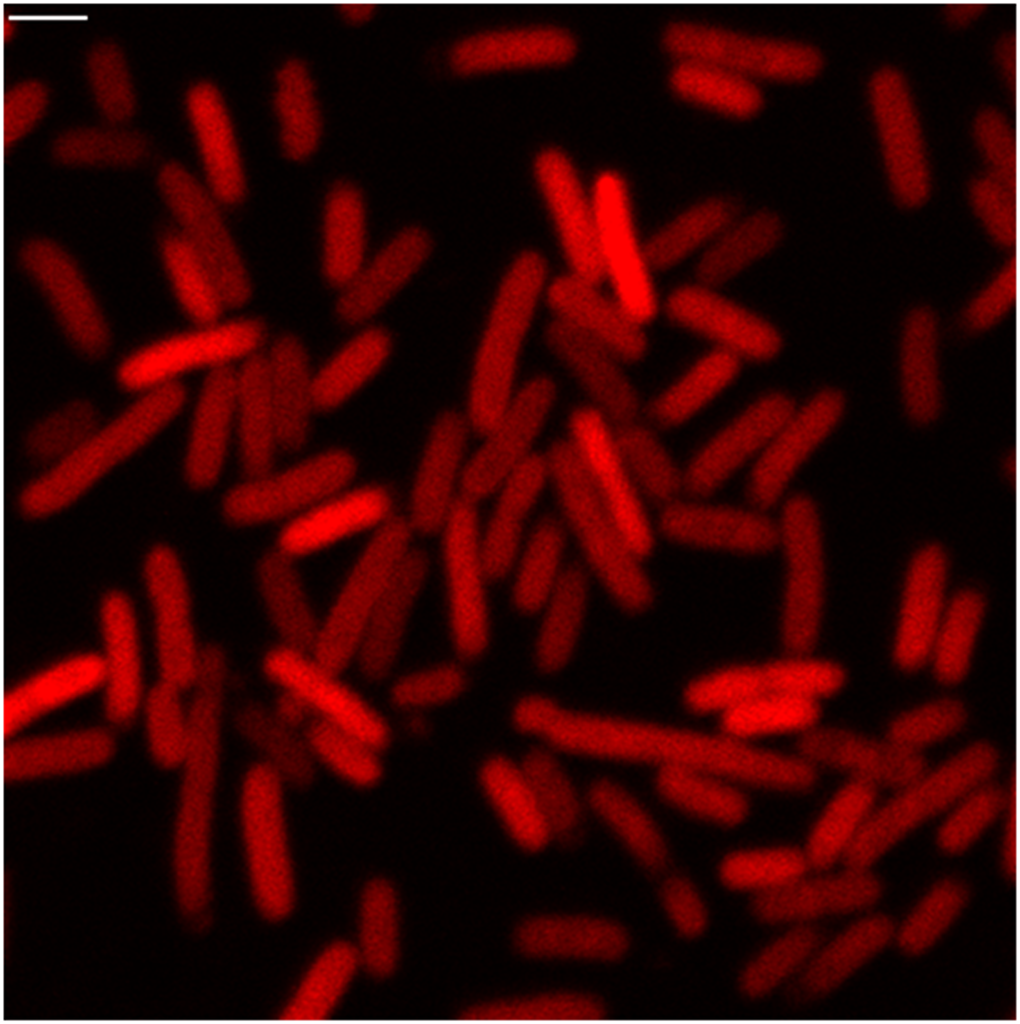

Supplement: FIG S1 [file mbio.01417-21-sf001.tif]

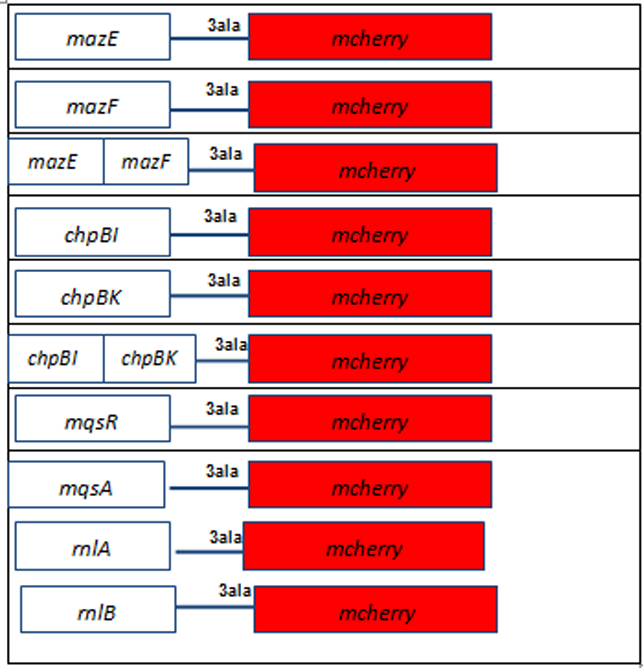

Supplement: FIG S2 [file mbio.01417-21-sf002.tif]

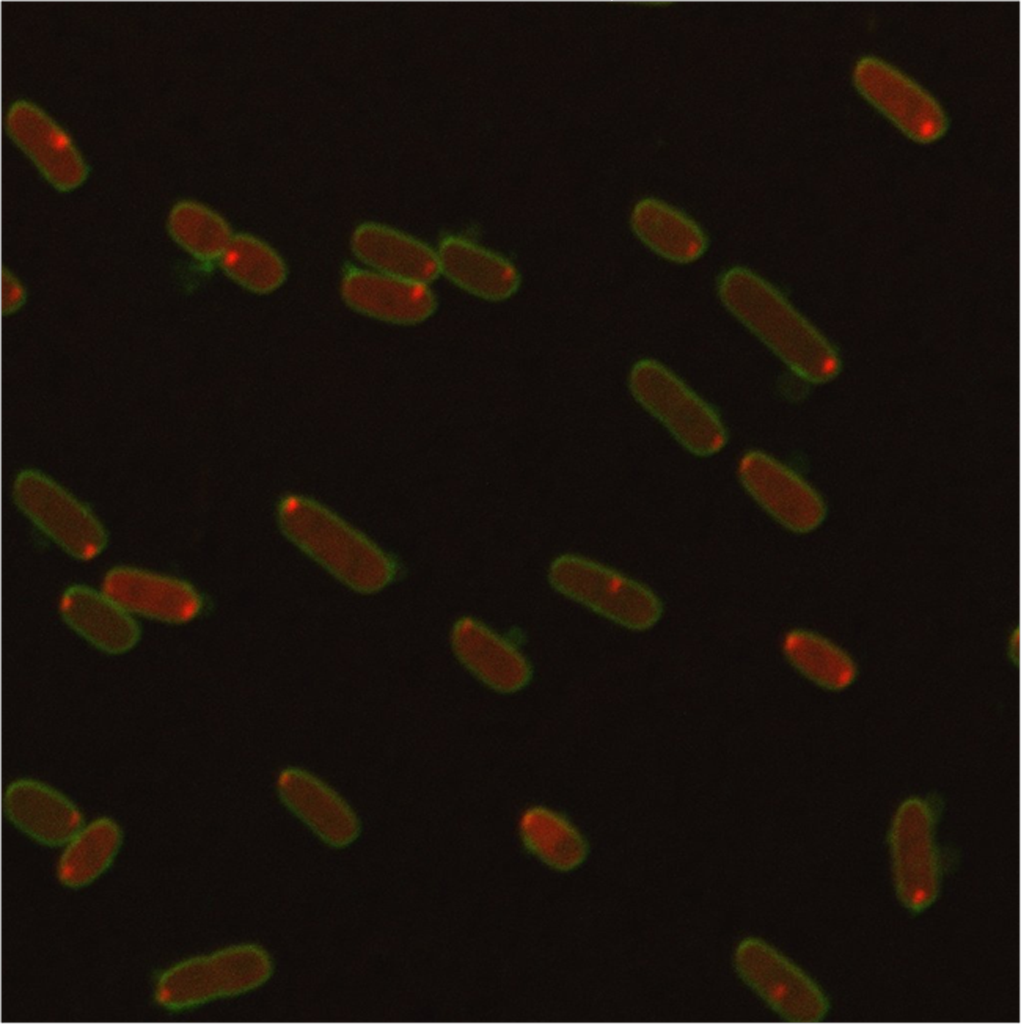

Supplement: FIG S3 [file mbio.01417-21-sf003.tif]

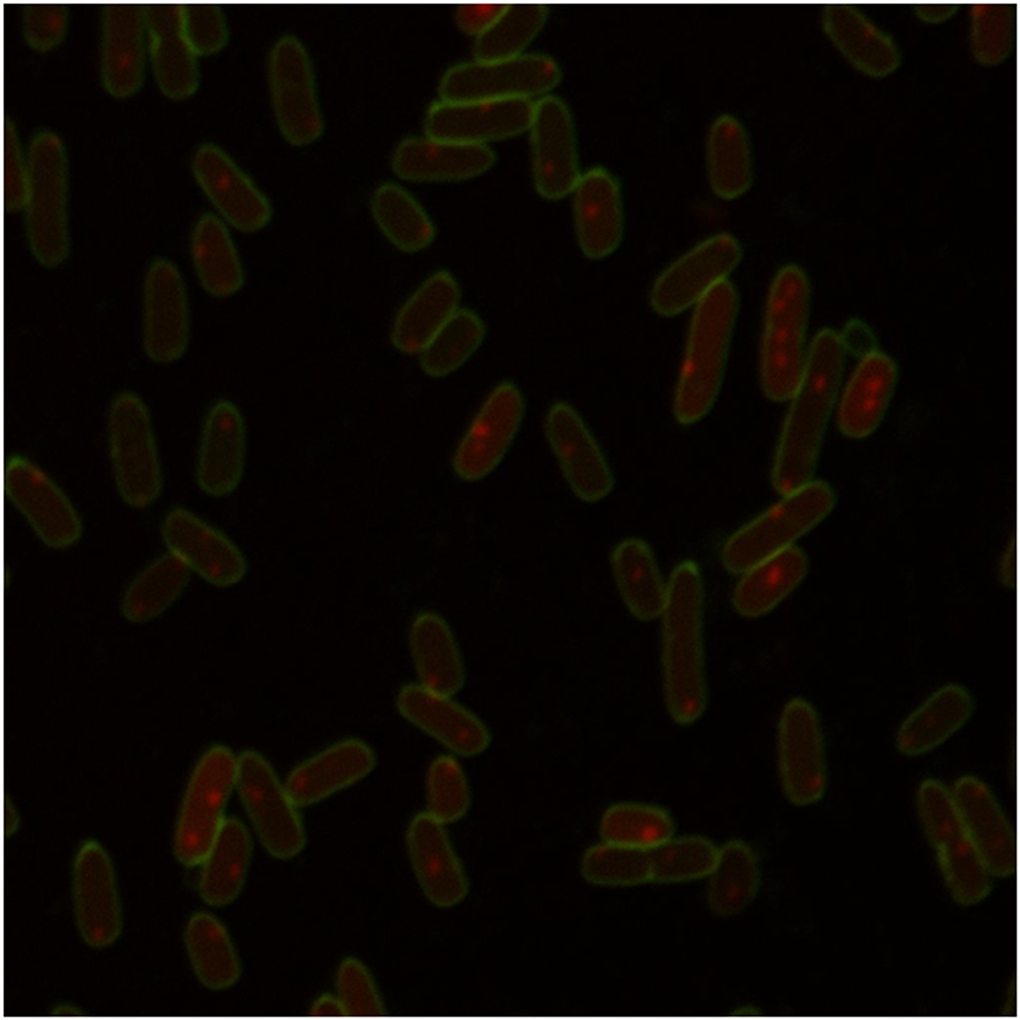

Supplement: FIG S4 [file mbio.01417-21-sf004.tif]

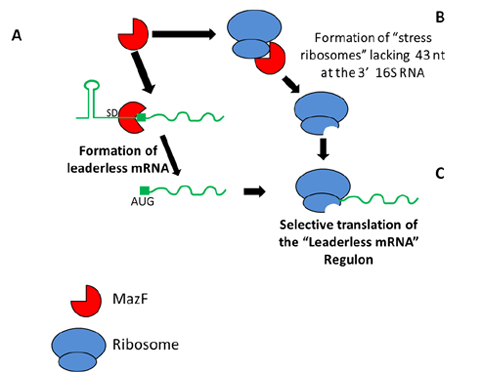

Supplement: FIG S5 [file mbio.01417-21-sf005.tif]
