# Supplementary material for: Intracellular Localization of the Proteins Encoded by Some Type II Toxin-Antitoxin Systems in Escherichia coli
Source: mBio. 2021 Aug 3;12(4):e01417-21. doi: 10.1128/mBio.01417-21 (PMC8406201; doi:10.1128/mBio.01417-21)
Supplement: Text S1 [file mbio.01417-21-t0001.docx]

**Text S1.** **Primers used in this work**

Primers for constructing pBAD18-*mazEF-mcherry* plasmid:

Forward:

5'-AAGAATTCATTAAAGAGGAAAGGGTTATGATCCACAGTAGCGTAAA-3'

Reverse:

5'-TTGAGCTCCCCAATCAGTACGTTAATTTT-3'

Primers for constructing pBAD18-*mazE-mcherry* plasmid:

Forward:

5'-AAGAATTCATTAAAGAGGAAAGGGTTATGATCCACAGTAGCGTAAA-3'

Reverse:

5'-TTGAGCTCCCAGACTTCCTTATCTTTCG-3'

Primers for constructing pBAD18-*mazF-mcherry* plasmid:

Forward:

5'-AAGAATTCATTAAAGAGGAAAGGGTTATGGTAAGCCGATACGTACC-3'

Reverse:

5'-TTGAGCTCCCCAATCAGTACGTTAATTT-3'

Primers for constructing pBAD18-*chpBIK-mcherry* plasmid:

Forward:

5'-AGAATTCATTAAAGAGGAAAGGGTTATGCGTATTACCATAAAAAG-3'

Reverse:

5'-TTGAGCTCTTCCACCACCGCCTGCAAGC-3'

Primers for constructing pBAD18-*chpBI-mcherry* plasmid:

Forward:

5'-AAGAATTCATTAAAGAGGAAAGGGTTATGCGTATTACCATAAAAAG-3'

Reverse:

5'-TTGAGCTCCCATATTTCGTCACCCGCAG-3'

Primers for constructing pBAD18-*chpBK-mcherry* plasmid:

Forward:

5'-AAGAATTCATTAAAGAGGAAAGGGTTATGGTAAAGAAAAGTGAATT-3'

Reverse:

5'-TTGAGCTCTTCCACCACCGCCTGCAAGC-3'

Primers for constructing pBAD18-*mqsR-mcherry* plasmid:

Forward:

5'-AAGAATTC ATTAAAGAGGAAAGGGTT ATGGAAAAAC GCACACCACA TCACGTTTG-3'

Reverse:

5'-TTGAGCTC CTTCTCCTTAAACGAGACGA-3'

Primers for constructing pBAD18-*mqsA-mcherry* plasmid:

Forward:

5'-AAGAATTC ATTAAAGGAGGTAAGGGTTATGAAATGTC CGGTTTGCCA-3'

Reverse:

5'-TTGAGCTCACGGATTTCATTCAATAGTT-3'

Primers for constructing pBAD18-*rnlA-mcherry* plasmid:

Forward:

5'-AAGAATTCATTAAAGGAGGTAAGGGTTATGACAATCA GGAGTTACAA-3'

Reverse:

5'-TTGAGCTCAACAATATATAAGTCCTTGATTATTCCCCACGC-3'

Primers for constructing pBAD18-*rnlB-mcherry* plasmid:

Forward:

5'- AAGAATTCATTAAAGGAGGTAAGGGTT TTGTTTGAAA TCACCGGAAT-3'

Reverse:

5'- TTGAGCTCAAAATCCATTGACAGGACTT-3'

Primers for constructing pBAD33-*mazE*

Forward:

5’-TGAGCTCATTAAAGGAGGTAAGGGTTATGATCCACAGTAG-3’

Reverse :

5’-AATCTAGAcgccgccgcCCCAATCAGTACGTTAATTTT-3’

Primers for constructing pBAD33-*mqsA*

Forward:

5'-TGAGCTCATTAAAGGAGGTAAGGGTTATGGAAAAACGCACACCACATACACGTTTG-3'

Reverse:

5'-AATCTAGACGCCGCCGCCTTCTCCTTAAACGAGACGA-3'

Primers for constructing pBAD33-*rnlB*

Forward:

5'- AAGAATTCATTAAAGGAGGTAAGGGTT TTGTTTGAAA TCACCGGAAT-3'

Reverse:

5'- TTGAGCTCAAAATCCATTGACAGGACTT-3'
