# Supplementary material for: Intracellular Localization of the Proteins Encoded by Some Type II Toxin-Antitoxin Systems in Escherichia coli
Source: mBio. 2021 Aug 3;12(4):e01417-21. doi: 10.1128/mBio.01417-21 (PMC8406201; doi:10.1128/mBio.01417-21)
Supplement: TABLE S1 [file mbio.01417-21-st001.docx]

**Table S1. Plasmids used in this work**

| Plasmid | Properties | Source |
| --- | --- | --- |
| pBAD18-*mcherry* | *mcherry* gene cloned into pBAD18 plasmid. Ara promoter, Ampicilin resistance, pBR332 ori. | Prof. Orna Amster-Choder's Lab  (1) |
| pBAD18-*mazEF-mcherry* | *mazEF* gene cloned into pBAD18-*mcherry* | This study |
| pBAD18-*mazE-mcherry* | *mazE* gene cloned into pBAD18-*mcherry* | This study |
| pBAD18-*mazF-mcherry* | *mazF* gene cloned into pBAD18-*mcherry* | This study |
| pBAD18-*chpBIK-mcherry* | *chpBIK* gene cloned into pBAD18-*mcherry* | This study |
| pBAD18-*chpBI-mcherry* | *chpBI* gene cloned into pBAD18-*mcherry* | This study |
| pBAD18-*chpBK-mcherry* | *chpBK* gene cloned into pBAD18-*mcherry* | This study |
| pBAD18-*mqsR -mcherry* | *mqsR* gene cloned into pBAD18-*mcherry* | This study* |
| pBAD18-*mqSA-mcherry* | *mqsA* gene cloned into pBAD18-*cherry* | This study* |
| pBAD18-*rnlA -mcherry* | *rnlA gene cloned into pBAD18-cherry* | This study* |
| pBAD18-*rnlB -mcherry* | *rnlB gene cloned into pBAD18-cherry* | This study* |
| pBAD33-*mazE* | *mazE* gene cloned into pBAD33 | This study |
| pBAD33-*mqsA* | *mqsA* gene cloned into pBAD33 | This study |
| pBAD33-*rnlB* | *rnlB* gene cloned into pBAD33 | This study |
| pSA1 | *mazF* gene cloned to PQE30 plasimd IPTG promoter AMP resistance pBR332 ori | Amitai,S. et. al, 2009  (2) |

1. Lopian L, Elisha Y, Nussbaum-Shochat A, Amster-Choder O. 2010. Spatial and temporal organization of the E. coli PTS components. EMBO J 29:3630–3645.

2. Amitai S, Kolodkin-Gal I, Hananya-Meltabashi M, Sacher A, Kulka HE. 2009. Escherichia coli MazF leads to the simultaneous selective synthesis of both “‘death proteins’” and “ ‘survival proteins.’” PLoS Genet 5.
